# Hypermethioninemia Leads to Fatal Bleeding and Increased Mortality in a Transgenic I278T Mouse Model of Homocystinuria

**DOI:** 10.3390/biomedicines8080244

**Published:** 2020-07-24

**Authors:** Insun Park, Linda K. Johnson, Allaura Cox, Brian R. Branchford, Jorge Di Paola, Erez M. Bublil, Tomas Majtan

**Affiliations:** 1 Section of Genetics & Metabolism, Department of Pediatrics, University of Colorado Anschutz Medical Campus, Aurora, CO 80045, USA; insun.park@ucdenver.edu; 2 Department of Pathology, University of Colorado Anschutz Medical Campus, Aurora, CO 80045, USA; linda.k.johnson@ucdenver.edu; 3 Section of Hematology/Oncology, Department of Pediatrics, University of Colorado School of Medicine, Aurora, CO 80045, USA; allaura.cox@cuanschutz.edu (A.C.); brian.branchford@childrenscolorado.org (B.R.B.); dipaolaj@wustl.edu (J.D.P.); 4 University of Colorado Hemophilia and Thrombosis Center, Aurora, CO 80045, USA; 5 Orphan Technologies Ltd., 8640 Rapperswil, Switzerland; erez.b@neopharmgroup.com

**Keywords:** homocysteine, methionine intake, thrombosis, homocystinuria, coagulation factors, bleeding

## Abstract

Severely elevated plasma homocysteine and methionine lead to thromboembolic events and strokes in homocystinuric (HCU) patients. Mouse models of HCU failed to exhibit prothrombotic phenotype, presumably due to lack of hypermethioninemia. We evaluated the impact of hypermethioninemia together with hyperhomocysteinemia on murine HCU phenotype and compared the efficacy of the current and novel therapies for HCU. High methionine intake decreased survival of I278T mice, which died from intestinal bleeding with hepatic and pancreatic failure. I278T mice on normal or increased methionine intake developed endothelial dysfunction, but paradoxically demonstrated delayed occlusion in an induced arterial thrombosis model. RNA-seq analysis suggested that expression of coagulation factor XI (FXI) is downregulated in livers of I278T mice. Indeed, plasma concentrations of FXI were decreased in I278T mice on normal diet and further reduced by increased methionine intake. Dietary methionine restriction normalized the observed phenotype. Similarly, treatment with OT-58, a novel enzyme therapy for HCU, corrected the phenotype in I278T mice regardless of their dietary methionine intake. Hypermethioninemia does not contribute to prothrombotic phenotype in murine HCU. Downregulation of FXI may contribute to the lack of prothrombotic tendency in I278T mice. Methionine restriction or treatment with OT-58 corrects vascular disease in the I278T mouse model of HCU.

## 1. Introduction

Elevated plasma total homocysteine (Hcy) causes endothelial dysfunction, increases thrombosis and is associated with carotid atherosclerosis, lacunar infarction and markedly increased risk of stroke in atrial fibrillation [1,2,3]. Severe hyperhomocysteinemia is a characteristic biochemical feature of classical homocystinuria (HCU), a rare autosomal recessive inborn error of sulfur amino acid metabolism caused by a deficiency in cystathionine beta-synthase (CBS) activity [4]. CBS is a pivotal enzyme in the conversion of an essential amino acid methionine (Met) to cysteine (Cys) [5]. Lack of CBS activity blocks condensation of Hcy with serine to cystathionine (Cth) and leads to toxic accumulation of Hcy, S-adenosylhomocysteine (SAH) and Met in plasma and tissues of affected individuals. Clinically, this biochemical imbalance results in skeletal and connective tissue disorders, impaired vision, cognitive impairment and vascular disease. Thromboembolism and stroke represent major clinical complications of the disease and are chiefly responsible for the premature death of HCU patients [4]. Treatment of HCU generally focuses on lowering plasma Hcy and symptomatic management of clinical exacerbations [6]. Dietary Met or protein restriction frequently combined with betaine supplementation constitutes the current standard of care. However, patients often find this diet difficult to follow, which exacerbates clinical complications [4,6]. Novel enzyme therapies, such as OT-58, are in development to provide more therapeutic options and to improve metabolic control, prevent clinical symptoms and lessen or remove entirely the dietary requirements [7,8,9]. OT-58, also known as 20NHS PEG-CBS or 20NHS PEG-htCBS C15S, is an engineered, recombinantly produced and chemically modified human CBS enzyme developed to reduce or normalize pathologically increased plasma Hcy [10]. OT-58 consists of the catalytic core of human CBS (residues 1-413) with a C15S mutation, which substantially decreases formation of higher order oligomers and soluble aggregates. Due to very short half-life of this catalytic core in circulation, the enzyme was chemically conjugated with polyethylene glycol (PEG) moieties. PEGylation with a linear 20 kDa N-hydroxysuccinimide-PEG (20NHS PEG) yielded the most reproducible pattern and showed the highest potency after repeated administration in a mouse model of HCU [7] and thus gave rise to 20NHS PEG-CBS candidate, which is undergoing clinical trials as OT-58.

Evaluation of the efficacy of new therapies for thromboembolism and vascular disease associated with HCU has been hindered by the availability of suitable mouse models, which would replicate this clinical symptom of the disease. Transgenic mouse CBS-deficient model expressing pathogenic human CBS I278T mutant under the control of a zinc-inducible promoter (the I278T model [11]) has been extensively studied in this regard. It was shown that severe hyperhomocysteinemia in I278T mice does indeed lead to endothelial dysfunction, however, it did not increase susceptibility to arterial or venous thrombosis [9,12,13]. Interestingly, I278T mice, unlike HCU patients, do not develop profound hypermethioninemia in addition to severely elevated plasma Hcy when maintained on standard rodent chow. It has been hypothesized that additional factors besides plasma Hcy contribute to the prothrombotic phenotype observed in humans and plasma Met was suggested as one such candidate prothrombotic factor. In this study, we developed hypermethioninemia in I278T mice by increasing their dietary Met intake and analyzed the effects of severely elevated plasma Hcy and Met on endothelial function and thrombosis. Furthermore, we evaluated the impact of methionine restriction with betaine supplementation as well as novel enzyme therapy for HCU on vascular phenotype of I278T mice.

## 2. Experimental Section

### 2.1. Chemicals and Test Compound

Unless stated otherwise, all materials were purchased from Sigma or Fisher Scientific. Human truncated cystathionine beta-synthase carrying C15S mutation PEGylated with linear 20kDa NHS ester PEG (20NHS PEG-CBS, OT-58) were prepared as described elsewhere [7].

### 2.2. Animals and Study Design

Procedures involving mice were performed at the University of Colorado Anschutz Medical Campus (UC AMC) under IACUC-approved protocol# 81. The UC AMC is an AAALAC-accredited (#00235; approved 25 July 2018), Public Health Service-assured (#D16-00171; expires 31 July 2023) and USDA-licensed (#84-R-0059; expires 7 November 2022) institution. A breeding pair of heterozygous transgenic I278T mice on the C57BL6 background was provided by Dr. Warren Kruger (Fox Chase Cancer Center, Philadelphia, PA, USA). Mice were propagated and genotyped at our facility as described previously [11]. Breeding pairs were maintained on extruded standard diet 2920X (Envigo, Madison, WI, USA) and water containing 25 mM ZnCl_2_ to induce transgene expression, and thus rescue the homozygous I278T pups from neonatal death. At week 4, homozygous I278T mice and their WT siblings were weaned, weighed, assigned into one of seven groups (Table 1) and received the first subcutaneous injection of vehicle (PBS) or OT-58 (10 mg/kg). Injections were administered three times a week (Monday, Wednesday, Friday) until termination of the study. At week 5, mice were switched to amino acid-defined diets with a reduced (0.5% Met, Envigo TD.110591, MRD), normal (4% Met, Envigo TD.170063, REG) or increased Met content (8.2% Met, Envigo TD.01084, HMD; see Supplementary Materials, Table S1 for detailed composition of the evaluated diets). In addition, I278T mice on the MRD diet received drinking water containing 2% (*w/v*) betaine hydrochloride instead of regular water. Mice were weighed weekly with weights used to calculate PBS or OT-58 injection volumes for the week. Beginning at week 6, blood samples were obtained once every 6 weeks to assess plasma sulfur metabolites. Samples were collected 24 h after the second injection in that week (i.e., on Thursdays). At week 18, mice were first analyzed by high-frequency ultrasound for non-invasive assessment of endothelium-dependent dilation. Mice were allowed to recover 1 day and afterwards, their in vivo thrombus formation time was determined using a ferric chloride (FeCl_3_)-induced carotid artery chemical injury thrombosis model. Shortly thereafter, the mice were exsanguinated and euthanized.

### 2.3. Plasma Collection and Analysis

A single-use lancet for submandibular bleeding was used for blood collection into BD Microtainer PST tubes with lithium heparin (Becto, Dickinson and Company, Franklin Lakes, NJ, USA). Tubes were then centrifuged at 10,000× *g* for 5 min, followed by transfer of plasma into 1.5 mL tubes and storage at −80 °C. Plasma sulfur amino acid metabolites were determined by stable-isotope-dilution liquid chromatography tandem mass spectrometry (LC–MS/MS) as described elsewhere [14].

### 2.4. Flow-Mediated Vasodilation (FMD)

Endothelium-dependent flow-mediated vasodilation was performed as described elsewhere [9,15]. Briefly, mice were anesthetized with isoflurane (3% induction and 1.5% maintenance). Body temperature was kept at 37 ± 1 °C by using a heated examination table that was also equipped with EKG electrodes and monitored using rectal thermometer probe. Hindlimbs were shaved and covered with warm ultrasound gel. The ultrasound probe was attached to a stereotactic holder and was manually aligned with the femoral vein visible at the upper inner thigh. We used the high-frequency, high-resolution Vevo 2100 imaging platform equipped with 30–70 MHz linear array micro-scan transducer (Fujifilm VisualSonics, Toronto, ON, Canada) to image the femoral arteries in mice. A vascular occluder (DocXS Biomedical Products, Ukiah, CA, USA) was placed around the lower limb to induce occlusion of the distal hindlimb as an ischemic trigger. Once a clear image of the vessel wall was obtained and baseline readings recorded, the experiment started by inflation of the vascular occluder for 5 min. Following hindlimb ischemia, the cuff was deflated and femoral artery diameter measurements were continuously recorded for 5 min at 30 s intervals. The femoral artery diameter in the acquired images was determined manually offline.

### 2.5. Carotid Artery Chemical Injury Thrombosis Model

The measurement was performed as described elsewhere [9,16]. Mice were anesthetized with intraperitoneal injection of a combination of ketamine (induction dose of 80–100 mg/kg, then 10–20 mg/kg/h for maintenance) and xylazine (induction dose of 8–16 mg/kg, then 1–2 mg/kg/h for maintenance) and body temperature was maintained at 37 ± 1 °C using a heating pad and monitored with a rectal thermometer probe. The right common carotid artery was exposed and baseline flow was monitored with a Doppler flow probe (Transonic Systems, Ithaca, NY, USA). For the artery injury, a 1 × 1 mm Whatman filter paper soaked in 6% FeCl_3_ solution was placed on the carotid artery for 3 min. Blood flow in the vessel was monitored and recorded for up to 20 min, using the ultrasound probe. The time to occlusion (TTO) was defined as the time between FeCl_3_ administration and lack of flow for 1 min. Blood was drawn from the inferior vena cava into a syringe flushed with 3.8% sodium citrate and processed to platelet-poor plasma by centrifugation at 2500× *g* for 5 min twice. At the end of the procedure, the mice were euthanized by cervical dislocation followed by bilateral thoracotomy.

### 2.6. Necropsy and Histopathology

Gross necropsies, tissue processing, staining and assessment were completed by the University of Colorado Cancer Center Research Histology Shared Resource. Sections of liver were collected and fixed in 30% formaldehyde and transferred to 70% ethanol until embedded into paraffin and processed on slides for hematoxylin-eosin (H&E) staining. Images were captured on an Olympus BX53 microscope using cellSens software and DP27 camera (Olympus, Waltham, MA, USA).

### 2.7. RNA-Seq Analysis

Total RNA was prepared from PBS-perfused livers of WT and I278T mice maintained on a standard extruded diet 2920X (Envigo, USA) by using RNeasy mini kit (Qiagen, Germantown, MD, USA) according to the manufacturer’s recommendations. Library preparation and sequencing was performed by Novogene (Sacramento, CA, USA). Differential expression analysis was performed using the DESeq R package (v1.18.0, Bioconductor project). DESeq provides statistical routines for determining differential expression in digital gene expression data using a model based on the negative binomial distribution. The resulting *p* values were adjusted using Benjamini and Hochberg’s approach for controlling the false discovery rate. Genes with an adjusted *p* < 0.05 were assigned as differentially expressed.

### 2.8. Quantification of FXI in Plasma

Coagulation factor XI was quantified in plasma samples from terminal bleedings using commercial mouse FXI ELISA kit (Aviva Systems Biology, San Diego, CA, USA) according to the manufacturer’s recommendations.

### 2.9. Statistical Analysis

Data are presented as mean ± standard error of the mean (SEM). Statistical analysis was conducted using ANOVA followed by Tukey’s multiple comparison test to determine significance. In some figures, significance is designated by asterisks or hash signs: */# for *p* < 0.05, **/## for *p* < 0.01 or ***/### for *p* < 0.001. In other figures, significance is designated by using letters at the top of the error bars and no letter indicates non-significance (or ns). Columns that are significantly different from each other (*p* < 0.05) are indicated by having a different letter. Two letters indicate that the column is not significantly different from the single letter designated columns.

## 3. Results

### 3.1. Higher Met Intake Leads to Increased Mortality of I278T Mice

**Figure 1:**
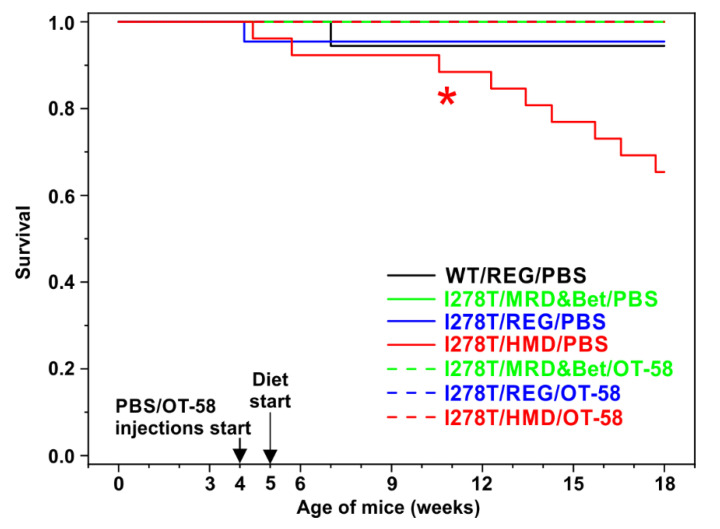
Survival of mice over the course of the study. Kaplan–Meier survival curves of WT controls on the REG diet and vehicle (PBS) injections (black) and I278T mice on the MRD&Bet/REG/HMD diet (green/blue/red) receiving subcutaneously either PBS (solid lines) or OT-58 (10 mg/kg, dashed lines). The asterisk (*) designates significantly lower survival rate of I278T mice on the HMD diet receiving PBS compared to WT controls (65% versus 94.4%, Log-rank test *p* = 0.0303).

In order to develop hypermethioninemia in I278T mice and to compare the efficacy of current and novel therapeutic approaches for HCU, I278T mice were randomized into six groups (Table 1). Administration of vehicle (PBS) or OT-58 via subcutaneous injections commenced at week 4 of age, while the new dietary regimen was initiated after weaning at week 5 of age. We observed that survival of I278T mice on the HMD diet injected with PBS was significantly decreased compared to WT controls and other groups of I278T mice (Log-rank test *p* = 0.0303) with 65% of mice alive at the end of the study compared to >94% in other cohorts (Figure 1). I278T mice receiving lower and normal amount of Met in the form of the MRD&Bet and REG diets, respectively, had similar survival to WT controls regardless of whether they received PBS injections or were treated with OT-58. Importantly, OT-58 treatment entirely rescued the survival of I278T mice on the HMD diet. These results suggest that higher Met intake leads to premature death of I278T mice.

### 3.2. Higher Met Intake Leads to Arrested Growth of I278T Mice

**Figure 2:**
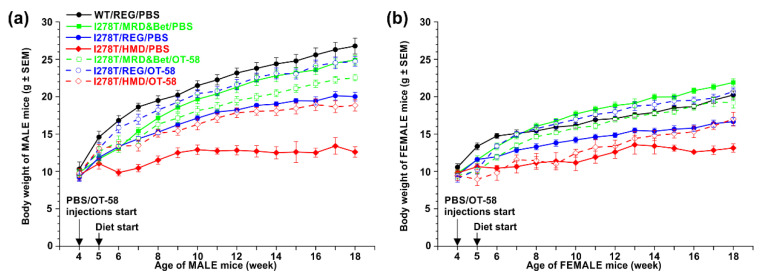
Comparison of the weight of WT and I278T mice over the course of the study. Males and females are shown separately in panels (**a**) and (**b**), respectively. WT controls were maintained on the REG diet and PBS injections (black), while I278T mice were on the MRD&Bet/REG/HMD diet (green/blue/red) receiving subcutaneously either PBS (closed symbols and solid lines) or OT-58 (10 mg/kg, open symbols and dashed lines). Data points represent average values and error bars show SEMs. Statistical significance was determined by using ANOVA, and for the sake of clarity, is mentioned only in the main text.

Reduced survival of I278T on the HMD diet and PBS injections was accompanied by arrested growth, as illustrated in a comparison of body weights of the studied mice (Figure 2). Body weights of males remained essentially unchanged from week 9 to the end of the study at week 18 (Figure 2a) and were significantly lower compared to all other groups (*p* < 0.01). Females handled the higher Met intake somewhat better with significantly lower body weights compared to other groups only in weeks 15–18 (Figure 2b; *p* < 0.05). Lower Met intake resulted in normal body weight of I278T mice compared to WT controls with females actually having higher body weight than the controls (weeks 12–17; *p* < 0.05). Normal Met intake caused lower body weight in I278T mice on the REG diet compared to WT controls (*p* < 0.05). Treatment with OT-58 significantly improved the body weight of I278T mice on the REG and HMD diets (*p* < 0.05), but not those on the MRD&Bet diet (Figure 2). Taken together, reduced survival of I278T mice on the HMD diet is accompanied by lower body weights, which is rescued by the MRD&Bet diet or treatment with OT-58.

### 3.3. I278T Mice on the HMD Diet Develop Severe Hypermethioninemia

**Figure 3:**
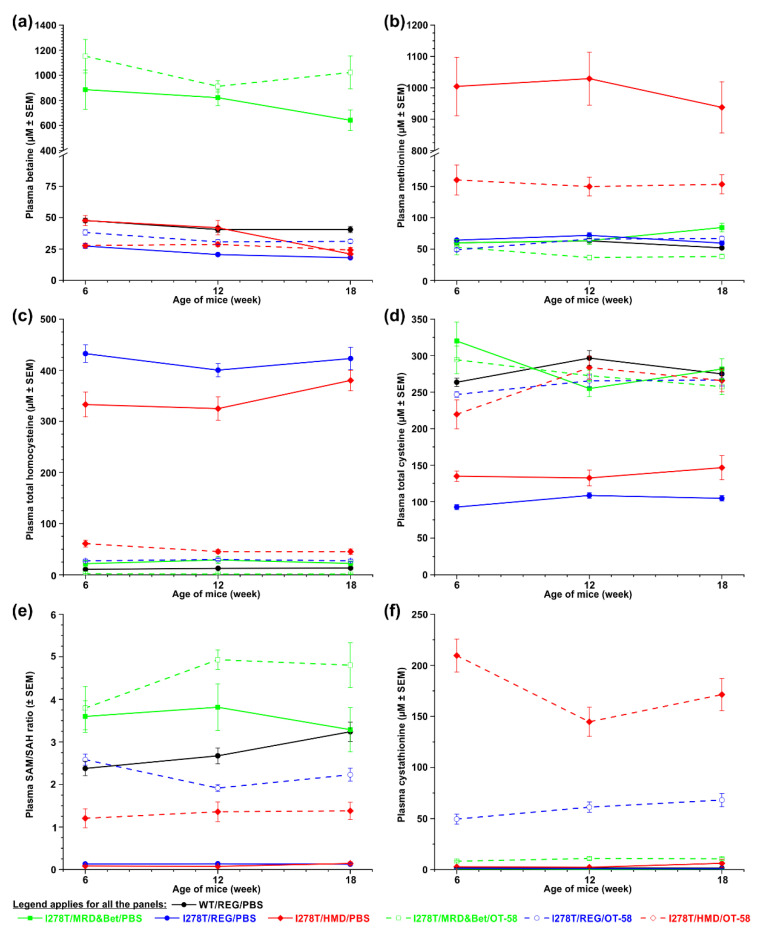
Time course of the selected sulfur metabolism-related plasma metabolites. The following metabolites were monitored in WT controls on the REG diet and PBS injections (black) as well as I278T mice maintained on the MRD&Bet/REG/HMD diet (green/blue/red) receiving subcutaneously either PBS (closed symbols and solid lines) or OT-58 (10 mg/kg, open symbols and dashed lines): (**a**) betaine, (**b**) methionine, (**c**) total homocysteine, (**d**) total cysteine, (**e**) SAM/SAH ratio and (**f**) cystathionine. Data points represent average values and error bars show SEMs. Statistical significance was determined by using ANOVA and, for the sake of clarity, is mentioned only in the main text. Note y axis breaks in panels (**a**) and (**b**) in order to provide better resolution.

Monitoring the plasma levels of sulfur metabolites during the course of the study showed that dietary intervention resulted in the desired outcomes. Specifically, drinking water containing 2% (*w/v*) betaine provided together with the MRD diet in order to mimic current standard of care for HCU patients resulted in a substantial 18- or 24-fold elevation of plasma betaine in the I278T mice compared to WT controls (783 or 1029 vs. 43 µM, *p* < 0.01; Figure 3a). More importantly, higher Met intake resulted in severe hypermethioninemia in I278T mice on the HMD diet and PBS injections (990.6 vs. 58.4 µM, i.e., a 17-fold increase compared to WT, *p* < 0.01; Figure 3b) in addition to severe hyperhomocysteinemia typical for the I278T model (340.6 vs. 12.2 µM, i.e., a 28-fold increase compared to WT, *p* < 0.001; Figure 3c). Consequently, plasma total cysteine was decreased 2-fold in this cohort compared to WT controls (137.9 vs. 278.4 µM, *p* < 0.001; Figure 3d). In addition, methylation potential determined by plasma S-adenosylmethionine to S-adenosylhomocysteine (SAM/SAH) ratio was 28-fold lower in this cohort compared to WT controls (2.8 vs. 0.1, *p* < 0.001; Figure 3e). As expected, I278T mice on the REG diet and PBS injections developed a similar biochemical profile except for plasma Met concentrations, which remained similar to those in WT controls (Figure 3b).

The MRD&Bet diet normalized plasma Met and total Cys (Figure 3b&d), substantially reduced plasma total Hcy to 2-fold higher than in WT controls (24.4 vs. 12.2 µM, *p* < 0.01; Figure 3c) and increased SAM/SAH ratio slightly above levels seen in WT controls (Figure 3e).

Treatment with OT-58 reduced plasma total Hcy concentrations in all treated I278T mice to 1.6, 28.2 and 50.6 µM on average in I278T mice on the MRD&Bet, REG and HMD diets, respectively, compared to WT controls on the REG diet (12.2 µM, *p* < 0.05; Figure 3c). Furthermore, OT-58 treatment substantially reduced hypermethioninemia induced by the HMD diet to 154.4 µM on average compared to 58.4 µM in WT controls (*p* < 0.05; Figure 3b). Plasma total Cys was fully normalized (Figure 3d) and the SAM/SAH ratio substantially improved (Figure 3e). As anticipated, administration of OT-58 markedly increased plasma Cth proportionally to Met intake to 10, 60 and 175 µM in I278T mice on the MRD&Bet, REG and HMD diets, respectively, compared to 1.7 µM in WT controls (*p* < 0.01; Figure 3f). The data illustrate that dietary regimes and OT-58 treatment resulted in anticipated and desired outcomes, and thus allowed the study of the effect of hypermethioninemia in addition to hyperhomocysteinemia on vascular phenotype in I278T mouse model of HCU in the absence or presence of the treatment (OT-58).

### 3.4. High Met Intake Caused Fatal Intestinal Hemorrhage and Organ Failure in I278T Mice

**Figure 4:**
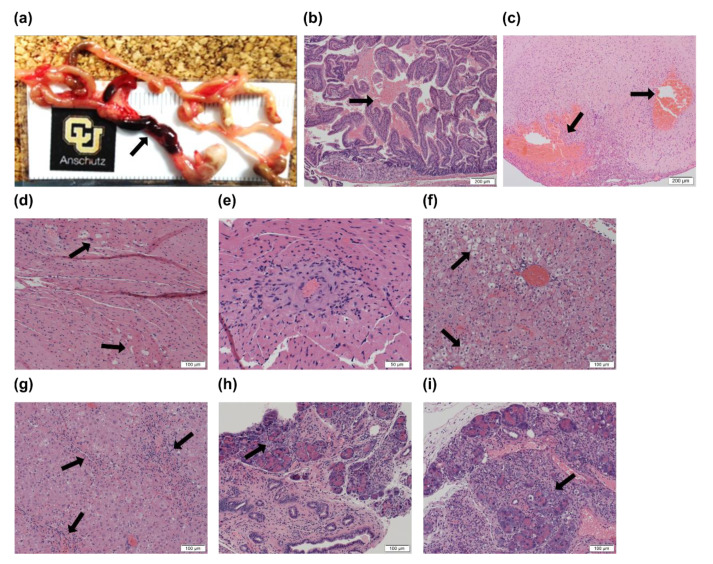
Necropsy and histopathology of deceased I278T mice on the HMD diet. Arrows point to the most common pathological findings identified in deceased I278T mice on the HMD diet and PBS injections: (**a**) blood-filled intestine, (**b**) blood cells in proximal duodenum, (**c**) caudal-ventral cerebral hemorrhage, (**d**) abnormal vacuoles in heart muscle, (**e**) inflamed heart vein, (**f**) microvesicular hepatosteatosis, (**g**) inflammation of liver parenchyma, (**h**) ductal hyperplasia of pancreas and (**i**) inflammation and destruction of normal pancreas parenchyma. Tissue sections were stained with H&E. Magnification varied and is indicated by a scale bar located in the bottom right corner of each panel.

In order to better understand the cause of premature death of I278T mice on the HMD diet, we performed necropsy and histopathological assessment of the deceased mice. Blood in the small intestine with a sharp demarcation between viable and blood-filled intestine (Figure 4a) and proximal duodenum with blood within the lumen (Figure 4b) were found consistently in the deceased I278T mice. Occasionally, pathological bleeding was found in other tissues, such as brain with caudal-ventral cerebral hemorrhage (Figure 4c). Heart muscle often contained abnormal vacuoles (Figure 4d) and heart vasculature showed signs of inflammation (Figure 4e). The liver showed many hepatocytes with condensed nuclei lacking noted nucleoli, substantial microvesicular steatosis (Figure 4f) and notable inflammation (Figure 4g) with occasional fibrosis. The pancreas of the mice was characterized by ductal hyperplasia, profound inflammation, fibrosis and destruction of normal parenchyma (Figure 4h,i). Exocrine function of the pancreas was likely affected. Together, the histopathology of the deceased I278T mice on the HMD diet suggests that liver and pancreas failure with hemorrhage rather than a thromboembolic event are likely the reasons for premature death of I278T mice suffering severe hypermethioninemia and hyperhomocysteinemia.

### 3.5. Normal and High Met Intakes Result in Endothelial Dysfunction, but Fail to Produce a Prothrombotic Phenotype in I278T Model of HCU

**Figure 5:**
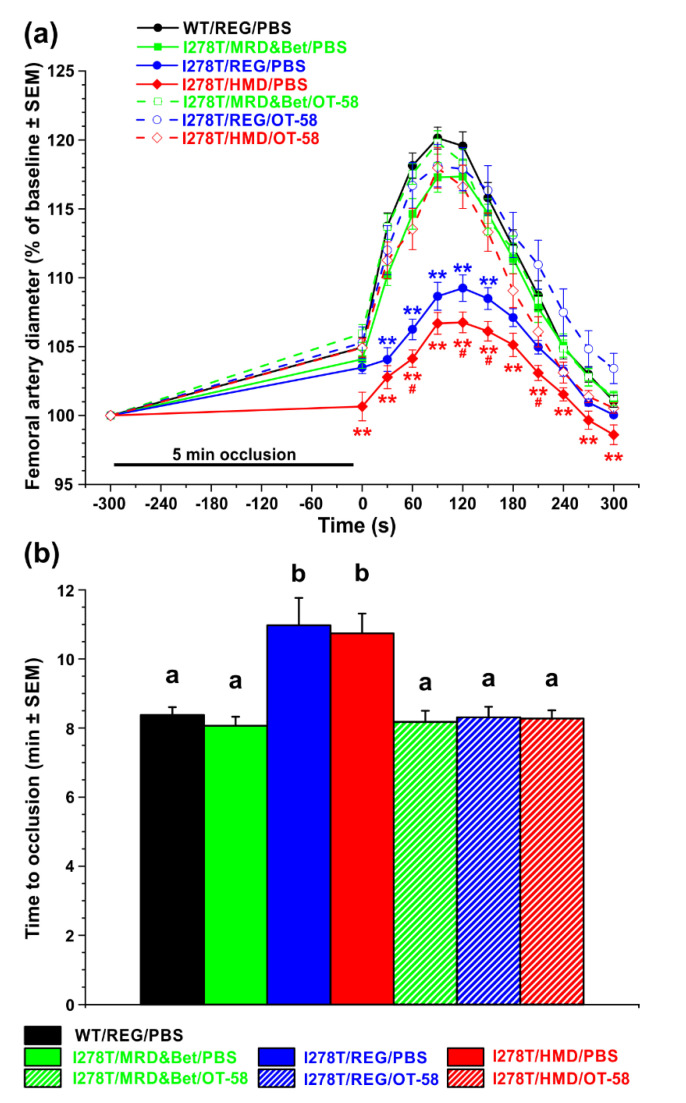
Evaluation of endothelial function and thrombotic phenotype. (**a**) Flow mediated vasodilation on femoral artery and (**b**) FeCl_3_-induced chemical injury to common carotid thrombosis model were used to evaluate endothelial function and thrombotic phenotype, respectively, in I278T mice maintained on the MRD&Bet/REG/HMD diet (green/blue/red) receiving subcutaneously either PBS (closed symbols and solid lines or solid fill) or OT-58 (10 mg/kg, open symbols and dashed lines or hatch fill) and compared to WT controls on the REG diet and PBS injections (black). (**a**) After obtaining a stable baseline level, 5 min occlusion with no blood flow induced reactive hyperemia characterized by temporal increase of artery diameter before returning to baseline. Data points represent average values and error bars show SEMs. Statistical significance was determined by using ANOVA. Asterisks denote significance compared to WT controls, while pound signs designate significance of I278T mice on the HMD diet and PBS injections compared to those on the REG diet and PBS injections. (**b**) Columns represent average time to stable occlusion in the carotid artery after chemical injury with SEMs as error bars. Significance was determined by ANOVA and is designated by letters above the error bars.

We evaluated endothelial function by using non-invasive FMD ultrasound method in the femoral artery of the studied mice, where brief total occlusion of the artery induced reactive hyperemia and changes in vascular hemodynamics dependent on and mediated by endothelial nitric oxide synthase. The diameter of femoral artery in the studied mice was 196 µM on average, which represented a baseline (100%). Following 5 min occlusion, dilation of the femoral artery peaked at 90 s after reperfusion with a 20.1% increase in diameter compared to baseline in WT controls and within 5 min it returned to normal level (Figure 5a). A similar profile was recorded for I278T mice on the MRD&Bet diet or those treated with OT-58 regardless of the diet. However, I278T mice on the REG and HMD diets injected with PBS showed substantial endothelial dysfunction with dilation peaking later at 120 s after reperfusion with only 9.2% and 6.8% increase in diameter compared to baseline, respectively (*p* < 0.05; Figure 5a).

Next, we examined whether severe hyperhomocysteinemia with hypermethioninemia influences the susceptibility to thrombosis using chemically-induced experimental thrombosis of the common carotid artery by 6% FeCl_3_ (Figure 5b). After chemical injury, stable occlusion formed at 8 min 22 s in WT controls. Similar to the results from the FMD study, I278T mice on the MRD&Bet diet or those treated with OT-58 regardless of the diet showed comparable time to stable occlusion. However, I278T mice on the REG and HMD diets injected with PBS showed delayed times to stable vessel occlusion of 10 min 58 s and 10 min 44 s, respectively (*p* < 0.05; Figure 5b). Taken together, HCU causes endothelial dysfunction and, paradoxically, decreases the susceptibility of I278T mice to the FeCl_3_-induced arterial thrombosis regardless of the absence or presence of hypermethioninemia. Dietary intervention with the MRD&Bet diet or treatment with OT-58 irrespective of dietary Met intake normalized the phenotype.

### 3.6. Substantially Decreased Plasma FXI Concentration Could Explain Internal Bleeding and Premature Death of I278T Mice on HMD Diet

**Figure 6:**
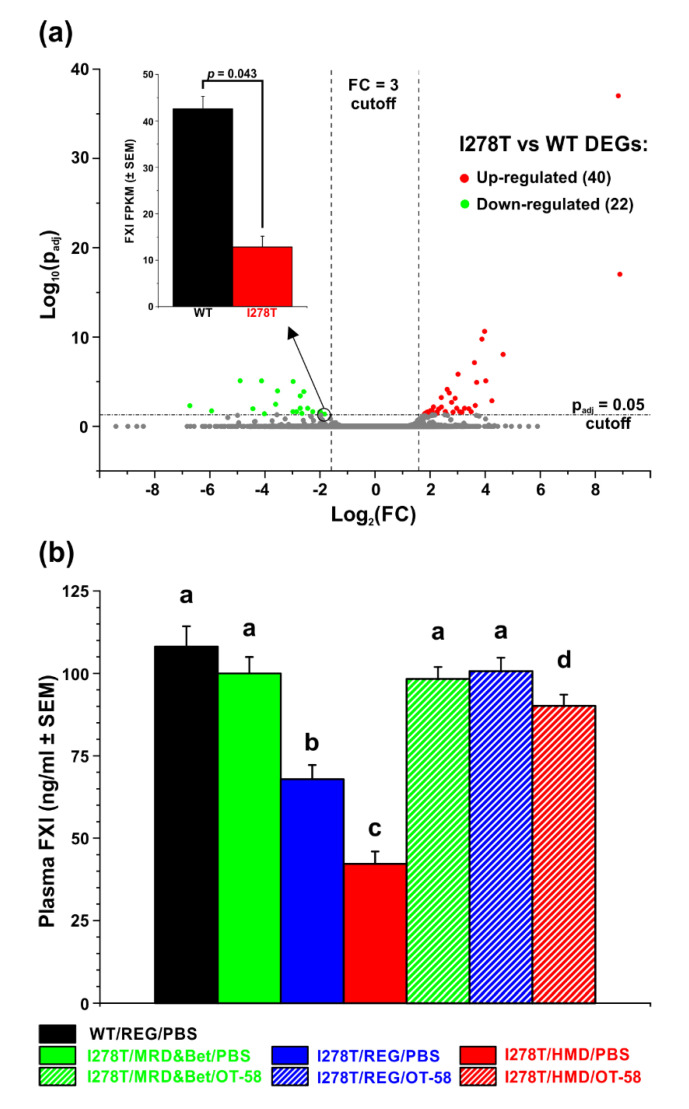
Differential expression of coagulation factor XI (FXI). (**a**) Volcano plot representation of differentially expressed genes in livers of I278T and WT mice maintained on standard rodent lab chow. Red and green points designate genes with significantly increased and decreased expression, respectively (*p* < 0.05). Inset shows comparison of actual RNA-seq reads for FXI transcripts in WT (black) and I278T (red) liver samples. (**b**) Quantification of FXI using ELISA in plasma samples of WT controls on the REG diet and PBS injections (black) and I278T mice maintained on the MRD&Bet/REG/HMD diet (green/blue/red) receiving subcutaneously either PBS (solid fill) or OT-58 (10 mg/kg, hatch fill). Columns represent average plasma FXI concentrations with SEMs as error bars. Significance was determined by ANOVA and is designated by letters above the error bars (*p* < 0.05).

Our findings of intestinal hemorrhage (Figure 4a,b), liver steatosis and inflammation (Figure 4f,g) in necropsies and lack of prothrombotic phenotype suggested that production of liver-synthesized coagulation factors might be impaired and responsible for our observations. Therefore, we evaluated differentially expressed genes in livers of I278T mice on the REG diet and compared them to WT controls on the same diet using RNA-seq (Figure 6a). Out of 62 differentially expressed genes, 40 and 22 genes were upregulated and downregulated, respectively, in I278T mice. Out of many coagulation factors produced in the liver, only one gene encoding coagulation factor XI was significantly downregulated in I278T mice (*p* = 0.043; Figure 6a inset). Subsequently, we assessed FXI concentrations in plasmas of the studied mice using ELISA assay (Figure 6b). Plasma FXI was found to be substantially decreased in I278T mice on either the REG (67.9 ng/mL; *p* < 0.001) or HMD diet (42.2 ng/mL; *p* < 0.001) and injected with PBS compared to WT controls (108.1 ng/mL). The MRD&Bet diet or OT-58 treatment normalized plasma FXI concentrations, although it remained slightly decreased in I278T mice on the HMD diet and treated with OT-58 (90.2 ng/mL, *p* < 0.05; Figure 6b). Taken together, our results suggest that severe hyperhomocysteinemia with or without hypermethioninemia disrupts production of liver-synthesized coagulation factor XI in a I278T mouse model of HCU.

## 4. Discussion

Thromboembolic events, particularly deep vein thrombosis and stroke, represent the major cause of morbidity and mortality in HCU patients [4,6]. However, the mechanism responsible for increased susceptibility to thrombosis in case of both mild and severe hyperhomocysteinemias remains unclear. In addition, the exact role of elevated plasma Hcy in this process is often debated because of contradictory results from several clinical trials focused on lowering plasma homocysteine and prevention of cardiovascular outcomes [17].

In this study, we evaluated the hypothesis that hypermethioninemia in addition to hyperhomocysteinemia is required for the prothrombotic phenotype in HCU and that it can be prevented by proper treatment. Genetic mouse models of CBS-deficient HCU are characterized by normal or slightly elevated plasma Met [11,18,19] in contrast to HCU patients, who often develop severe hypermethioninemia [4,18]. The risk for vascular complications in HCU patients is significantly reduced, but not entirely eliminated by dietary management of the disease despite the fact that plasma Hcy levels often remain moderately elevated [20,21]. Mildly increased plasma total Hcy is associated with high risk of thrombosis and stroke in non-HCU population [17].

In order to elevate normal plasma Met in I278T mice, we roughly doubled Met content in the diet compared to standard rodent chow. Indeed, we achieved almost 1000 µM plasma Met in I278T mice on the HMD diet (i.e., a 17-fold increase compared to WT mice), which is now in the range seen in unmanaged HCU patients or patients on betaine supplementation [4,18]. Increased Met intake resulted in a reduced survival and growth arrest of the I278T mice. Necropsy and histopathology revealed that deceased mice suffered from liver and pancreatic failure and likely died due to intestinal hemorrhage. While livers of I278T mice were shown to have mild steatosis, increased inflammation and oxidative stress, but no detectable hepatocyte damage [8,11], profound microvesicular hepatosteatosis and inflammation, fibrosis and destruction of normal parenchyma in both liver and pancreas can clearly be attributed to higher Met intake. Similar changes in liver were observed in 18 day-old complete CBS knock-out (KO) pups [22] and moribund surviving KO mice, in which liver injury was correlated by >30-fold elevation of liver ALT [18].

Here we showed that PBS-injected I278T mice on the REG or HMD diet suffer from endothelial dysfunction, but they showed delayed rather than accelerated thrombosis after chemical injury of the carotid artery. This paradoxical and rather unexpected finding, which is in contrast to the propensity to thromboembolic events in HCU patients, has been observed previously in I278T mice [9,12], but not in another transgenic mouse model of HCU lacking mouse CBS activity, but expressing human CBS WT gene under control of human CBS promoter (HO mouse) [18]. The main difference between these transgenic models is the amount of residual CBS activity and expression of the transgenes. While livers of I278T mice have roughly 2% [11], HO mice liver extracts exhibited 5% of the enzyme activity of that found in WT controls [19]. This seemingly negligible difference in residual CBS activity resulted in 40% lower plasma total Hcy concentration (243 versus 407 µM), lack of liver steatosis or fibrosis, and normal bone mineralization and fat content in HO compared to I278T mice [19,23]. Expression of the human CBS I278T transgene is induced by the divalent metal ions, such as zinc, received from diet or water, while the HO mice constitutively express human CBS WT transgene. On the other hand, unlike I278T mice, HO mice exhibited a prothrombotic phenotype using tail bleeding assay, which was significantly ameliorated by betaine supplementation [19]. These data suggest that the absence of liver impairment in HO mice in contrast to I278T or KO models may better represent the major clinical symptom of HCU. Although rare, liver failure associated with hypoalbuminemia, elevated bilirubin and transaminases and abnormal prothrombin time occurs in HCU [24].

Gastrointestinal hemorrhages are common complications in patients with advanced liver disease and are directly linked to liver function as many factors in the coagulation pathways are synthesized in liver [25,26]. To understand the absence of accelerated thrombosis in I278T mice and elucidate intestinal hemorrhage in deceased I278T mice on the HMD diet, we performed RNA-seq analysis of liver tissue and found that expression of FXI was significantly decreased. Subsequent ELISA confirmed decreased plasma FXI concentrations in I278T mice on the REG or HMD diet and PBS injections. However, previous hemostasis evaluation of coagulation cascade in I278T mice did not show any differences in prothrombin time, activated partial thromboplastin time or plasma fibrinogen levels compared to WT controls [12,13]. Previously, we found normal levels of ALT or AST markers of liver injury in I278T mice on standard rodent chow [8]. Together with data presented here it seems that a 37% decrease of plasma FXI in I278T on the REG diet does not lead to premature death, but results in a delayed arterial occlusion, which is contrary to HO or high Met/low fat diet-induced hyperhomocysteinemia mouse models [19,27]. Although the liver has a remarkable ability to regenerate and recover from injury [28], the presence of constant pressure in the form of missing CBS activity and additional stressors, such as increased Met intake, likely further exacerbated liver disease and dysfunction in I278T mice. Indeed, a 61% decrease of plasma FXI was associated with significantly reduced survival of I278T mice on the HMD diet and likely resulted in the intestinal hemorrhages observed in the deceased mice in this cohort. Liver dysfunction seems to be the critical factor affecting prothrombotic phenotype in HCU models. Unlike HO mice, KO mice suffer from severe liver injury characterized by steatosis, mild fibrosis and severe elevation of ALT activity in plasma [18,22]. More importantly, it was shown that tail bleeding time of a few surviving KO mice was not significantly different compared to WT controls regardless of the presence or absence of betaine supplementation [18]. HCU patients often show deficiency in antithrombin III (AT-III) and coagulation factors FVII, FIX, FX and FXI without elevation in thrombin-antithrombin III complexes, prothrombin fragment F1+2, fibrin monomer and d-dimers [29]. Therefore, sustained activation of coagulation has been excluded as the cause of low levels of coagulation factors. In addition, incubation of normal plasma with up to 400 µM Hcy did not alter the activity of the above-mentioned coagulation factors [29]. Taken together, deficient liver synthesis of coagulation factors most likely explains the observed low levels. Therefore, contrary to our hypothesis, hypermethioninemia in addition to hyperhomocysteinemia does not induce prothrombotic phenotype, but rather impairs liver function, which leads to the decreased synthesis of coagulation factors.

Treatment of HCU in I278T mice, either by dietary management as mimicked here by the MRD diet and betaine supplementation or by administration of OT-58 enzyme therapy, resulted in normal survival, an improved plasma metabolic profile and normalization of endothelial dysfunction as well as time to occlusion after carotid injury. Treatment with OT-58 improved the weight of I278T mice on the REG and HMD diets, but not on the MRD&Bet diet. This observation can be explained by OT-58 outcompeting betaine for their common substrate Hcy. While betaine conserves sulfur in the methionine cycle by promoting the conversion of Hcy back to Met [18], OT-58 irreversibly removes Hcy from the methionine cycle by condensation with serine into Cth. Thus, under severe Met restriction (i.e., 8- to 10-fold lower Met content than in the REG or standard rodent chow, respectively), betaine supplementation promoted the growth and weight gain of I278T mice, while OT-58 fully normalized plasma total Hcy levels. On the other hand, OT-58 treatment substantially reduced plasma Hcy in I278T mice on the REG or HMD diet. In the presence of high Met intake, OT-58 reduced profound hypermethioninemia, although plasma Met remained elevated roughly 3-fold compared to WT controls. Similarly, betaine treatment of HCU patients, reduces plasma Hcy and helps to prevent vascular complications, but often results in hypermethioninemia [30]. These results again point to the conclusion that hypermethioninemia in addition to hyperhomocysteinemia is not required for prothrombotic phenotype in HCU. The remarkable efficacy of OT-58 in decreasing plasma Hcy under variable Met intake is highly clinically relevant, as the compliance of HCU patients with dietary regimes is often poor. The presence of OT-58 in the bloodstream could therefore protect HCU patients from spikes in plasma Met and Hcy in periods of dietary non-compliance or lessen dietary Met restriction, thus potentially leading to decreased exacerbation of clinical complications and improved quality of life.

In summary, we found that hypermethioninemia in addition to hyperhomocysteinemia, as often seen in HCU patients, does not elicit a prothrombotic phenotype in I278T mice. Instead, an increased Met intake by I278T mice increased mortality, compromised liver function and impaired synthesis of coagulation factors, such as FXI, which eventually resulted in fatal intestinal hemorrhage. Treatment with novel enzyme therapy OT-58 prevented endothelial dysfunction and normalized thrombosis under highly different Met intakes despite imperfect plasma metabolic profiles. These findings contribute to our understanding of vascular phenotype in HCU and the role of increased plasma Met in the synthesis of coagulation factors and hemostasis. Furthermore, our observations may provide insights into dietary management and treatment of HCU patients once enzyme therapies in development or clinical trials, such as OT-58, receive regulatory approval and reach patients.

## Figures and Tables

**Table 1:** Study design.

Group ^1^	Genotype	Diet	Injections	Number of Mice (Males/Females)
1	+/+ (WT)	REG	PBS	18 (9M & 9F)
2	-/- (I278T)	MRD&Bet	PBS	22 (10M & 12F)
3	-/- (I278T)	REG	PBS	26 (15M & 11F)
4	-/- (I278T)	HMD	PBS	18 (8M & 10F)
5	-/- (I278T)	MRD&Bet	OT-58	17 (8M & 9F)
6	-/- (I278T)	REG	OT-58	18 (10M & 8F)
7	-/- (I278T)	HMD	OT-58	17 (8M & 9F)

^1^ All mice were raised on standard rodent chow (Envigo 2920X) and water containing 25 mM ZnCl_2_ until day 28, then switched to amino acid-defined diets as indicated. Beginning from day 21, mice received subcutaneous injections of either vehicle (PBS) or enzyme therapy (OT-58; 10 mg/kg) three times a week (Monday, Wednesday, Friday). I278T mice on the MRD diet received drinking water containing 2% (*w/v*) betaine hydrochloride as indicated. Blood was collected once every 6 weeks, beginning from week 6, for measurements of plasma metabolites.

## Supplementary Materials

Supplementary Materials can be found at https://www.mdpi.com/2227-9059/8/8/244/s1.
