# Supplementary material for: Hypermethioninemia Leads to Fatal Bleeding and Increased Mortality in a Transgenic I278T Mouse Model of Homocystinuria
Source: Biomedicines. 2020 Jul 24;8(8):244. doi: 10.3390/biomedicines8080244 (PMC7459533; doi:10.3390/biomedicines8080244)
Supplement: Supplementary file 1 [file biomedicines-08-00244-s001.pdf]

Supplementary Table S1. Amino acid-defined diets used in the study.

Component (g/kg)	MRD (TD.110591)	REG (TD.170063)	HMD (TD.01084)
L-Alanine	3.5	3.5	3.5
L-Arginine.HCl	12.1	12.1	12.1
L-Asparagine	6.0	6.0	6.0
L-Aspartic Acid	3.5	3.5	3.5
L-Cystine	3.5	3.5	3.5
L-Glutamic Acid	40.0	36.5	40.0
Glycine	23.04	23.04	23.3
L-Histidine.HCl, monohydrate	4.5	4.5	4.5
L-Isoleucine	8.2	8.2	8.2
L-Leucine	11.1	11.1	11.1
L-Lysine.HCl	18.0	18.0	18.0
L-Methionine	0.5	4.0	8.2
L-Phynylalanine	7.5	7.5	7.5
L-Proline	3.5	3.5	3.5
L-Serine	3.5	3.5	3.5
L-Threonine	8.2	8.2	8.2
L-Tryptophan	1.8	1.8	1.8
L-Tyrosine	5.0	5.0	5.0
L-Valine	8.2	8.2	8.2
Sucrose	353.14	353.14	344.98
Corn Starch	150.0	150.0	150.0
Maltodextrin	150.0	150.0	150.0
Soybean Oil	80.0	80.0	80.0
Cellulose	30.0	30.0	30.0
Mineral Mix (AIN-93M-MX)	35.0	35.0	35.0
Calcium Phosphate, monobasic, monohydrate	8.2	8.2	8.2
Vitamin Mix (AIN-93-VX)	19.5	19.5	19.5
Choline bitartarate	2.5	2.5	2.7
TBHQ (antioxidant)	0.02	0.02	0.02
**Summary nutrient information (% by weight / % kcal from)**			
Proteins	16.5 / 16.4	14.9 / 15.1	17.3 / 17.2
Carbohydrates	65.7 / 65.6	65.7 / 66.7	64.9 / 64.8
Fat	8.0 / 18.0	8.0 / 18.3	8.0 / 18.0
Energy density (Kcal/g)	4.0	3.9	4.0
